# Recognition and Repetition Counting for Local Muscular Endurance Exercises in Exercise-Based Rehabilitation: A Comparative Study Using Artificial Intelligence Models

**DOI:** 10.3390/s20174791

**Published:** 2020-08-25

**Authors:** Ghanashyama Prabhu, Noel E. O’Connor, Kieran Moran

**Affiliations:** 1 Insight SFI Research Centre for Data Analytics, Dublin City University, Dublin 9, Ireland; noel.oconnor@dcu.ie (N.E.O.); kieran.moran@dcu.ie (K.M.); 2 School of Electronic Engineering, Dublin City University, Dublin 9, Ireland; 3 Manipal Institute of Technology, MAHE, Manipal 576104, India; 4 School of Health and Human Performance, Dublin City University, Dublin 9, Ireland

**Keywords:** exercise-based rehabilitation, local muscular endurance exercises, deep learning, AlexNet, multi-class classification, INSIGHT-LME dataset

## Abstract

Exercise-based cardiac rehabilitation requires patients to perform a set of certain prescribed exercises a specific number of times. Local muscular endurance exercises are an important part of the rehabilitation program. Automatic exercise recognition and repetition counting, from wearable sensor data, is an important technology to enable patients to perform exercises independently in remote settings, e.g., their own home. In this paper, we first report on a comparison of traditional approaches to exercise recognition and repetition counting (supervised ML and peak detection) with Convolutional Neural Networks (CNNs). We investigated CNN models based on the AlexNet architecture and found that the performance was better than the traditional approaches, for exercise recognition (overall F1-score of 97.18%) and repetition counting (±1 error among 90% observed sets). To the best of our knowledge, our approach of using a single CNN method for both recognition and repetition counting is novel. Also, we make the INSIGHT-LME dataset publicly available to encourage further research.

## 1. Introduction

Cardiovascular disease (CVD) is the leading cause of premature death and disability in Europe and worldwide [1]. Exercise-based cardiac rehabilitation is a secondary prevention program which has been shown to be effective in lowering the recurrence rate of CVD and improves the health related quality of life [2,3,4,5,6]. Exercise-based cardiac rehabilitation is long-term exercise maintenance by patients attending community-based rehabilitation programs or through home-based exercise self-monitoring programs. However, a significant challenge is that uptake and adherence of community-based cardiac rehabilitation are very low, whereby only 14% to 43% of cardiac patients participate in rehabilitation programs [7,8]. Key reasons for lower participation include a lack of disease-specific rehabilitation programs, long travel times and scheduling issues to such programs [9]. In addition, patients may have low self-efficacy because of a perception of poor body image or poor exercise technique [9]. A potential solution to these challenges is the development of a technological platform for assessing exercise movement that can motivate the user to engage with exercise-based cardiac rehabilitation and enable them to do so in any environment (“anywhere exercising”).

Technology advances in sensor manufacturing and micro-miniaturization have resulted in low-cost micro-sensor wearable devices that are capable of effective lossless streaming and/or storing translatory and rotary movement information for further processing [10,11]. Machine learning (ML) and deep learning are artificial intelligence methods that employ statistical techniques to learn underlying hidden distributions from observed data. The application of ML methods to study data from human movements and activities to detect and understand these activities are referred to as human activity recognition (HAR). In recent years, many ML and deep learning-based models have been used along with wearable sensors in the assessment of human movement activities in many domains including: health [11], recreation activities [12], musculoskeletal injuries or diseases [13], day-to-day routine activities (e.g., walking, jogging, running, sitting, drinking, watching TV) [11,14,15,16,17,18,19,20,21], sporting movements [22] and exercises [23,24,25,26,27]. The ML models used for exercise recognition have predominantly used multiple wearable sensors [28,29,30,31], specifically in the areas of free weight exercise monitoring [32], the performance of lunge evaluation [24], limb movement rehabilitation [33], intensity recognition in strength training [34], exercise feedback [24], qualitative evaluation of human movements [28], gym activity monitoring [29], rehabilitation [23,25,33,35] and indoor-based exercises for strength training [36]. However, the use of multiple sensors is far from ideal in practice because of cost, negative aesthetics and reduced user uptake [17]. Studies [8,15,17,19] on the usage of wearable sensors, either phone-based or using inertial measurement units, have shown that CVD patients (67~68%) have an interest in single sensor-based cardiac rehabilitation [8]. Exercise-based applications, using single sensors, include recognizing day-to-day activities [26,37,38,39,40,41], and multiple complex exercises [23,26,27] or single exercises such as lunges [24] and squats [42], as well as repetition counting [27,43,44]. Therefore, in our research, we will use a single wrist-worn inertial sensor for exercise recognition and repetition counting.

In an ideal scenario, people would undertake a variety of exercise programs, either specifically prescribed or based on personal preference, that suits their goals and that allows them to avoid exercise associated with comorbidities (e.g., arthritis of the shoulder). In this scenario of “exercising anywhere” or self-responsible home-based exercising, it is extremely important that they receive feedback on the exercises to help them track their progress and stay motivated. However, two key challenges are presented with this approach. First, it is important to be able to automatically recognize which exercises are being completed, and secondly, once recognized to provide the number of repetitions as quantitative feedback on the amount of exercise performed to build the user’s competence and confidence. This would also allow people to complete elements of their training program disbursed over the day in any environment, as recommended by the American College of Sports Medicine [45]. For example, someone could complete different exercises in-home or in the workplace. To date, the vast majority of HAR studies detailed above have used traditional ML approaches such as decision trees, Naive Bayes, random forest, perceptron neural networks, k-nearest neighbor and support vector machines. There is, however, a growing interest in the potential use of deep learning methods in the field of activity recognition mainly using CNN [27,46,47,48,49] and recurrent models [47,50]. A small number of studies [46,47,49,51] have shown the significant advantage of using deep learning models in the general area of HAR. However, very few studies [23,25,27,30] appear to have used deep learning models in exercise recognition and repetition counting, and where employed they use multiple CNN models for the repetition counting task. To the best of our knowledge, there are no works reported using a single deep CNN model for exercise recognition and for repetition counting. The use of a single model for repetition counting is attractive as it eliminates the need for an exercise specific repetition counter and reduces the dependency on the total number of resources required in repetition computation. No other studies appear to have studied a wide number of exercises, and none specifically for CVD rehabilitation through LME exercises. In addition, no studies have undertaken a comparative study of using traditional ML methods and state-of-the-art CNN methods to identify the best possible method for exercise recognition and repetition counting.

We focus our study on exercise recognition and repetition counting using a single wrist-worn inertial sensor for 10 local muscular endurance (LME) exercises that are specifically prescribed in exercise-based CVD rehabilitation, with the following goals:

- To undertake a comparative analysis between different traditional supervised ML algorithms and a deep CNN model based on the state-of-the-art architecture and to find the best model for exercise recognition.
- To have a comparative analysis of traditional signal processing approach with a single deep CNN model based on the state-of-the-art architecture and to find the best model for exercise recognition.

As the novelty of this work, we claim the following novel contributions. First, we propose the use of a single CNN model for the repetition counting task of a wide range of exercises. Secondly, we are making the LME exercise dataset (INSIGHT-LME Dataset) publicly available (https://bit.ly/30UCsmR) to encourage further research on this topic.

## 2. Materials and Methods

### 2.1. Data Acquisition (Sensors and Exercises)

Currently, there exist no publicly available data-sets with a single wrist-worn sensor for endurance-based exercises that are commonly prescribed in cardiovascular disease rehabilitation (CVD) programs. Therefore, we collected a new data set of LME exercises prescribed in CVD rehabilitation program for balancing and muscle strengthening. In the data collection process, consenting participants performed the ten LME exercises in two sets (constrained set and unconstrained set) and some common movements which were observed by any exerciser in between two exercises. The constrained set of exercises involves participants performing the exercises while observing demonstrative videos and following the limb movement actions relatively synchronous with the demonstrator in the video. The unconstrained set of exercises involved participants performing the set of LME exercises without the assistance of demonstrative videos. Inclusion of the non-exercise movements was essential so that the built models can distinguish the actions corresponding to the exercise movements from that of non-exercise movements. The data set was then used for training, validating and testing different ML and deep neural network models.

#### 2.1.1. Sensor Calibration

**Figure 1:**
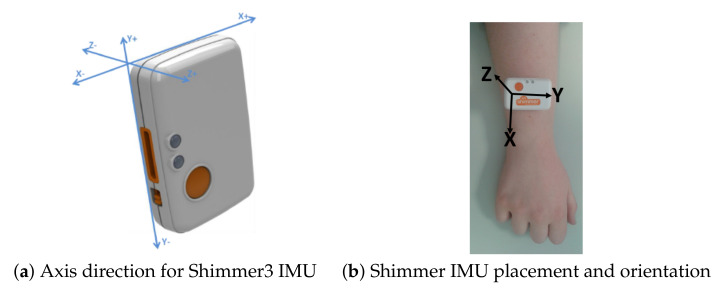
Shimmer3 IMU, axis direction, sensor placement and sensor orientation on the right wrist.

Sensor calibration is a method of improving the sensor unit’s performance to get a very precise and accurate measurement. The Shimmer3 (Figure 1a) inertial measurement unit (IMU) is a light-weight wearable sensor unit from Shimmer (http://www.shimmersensing.com). Each IMU comprises of a 3 MHz MSP430 CPU, two 3D accelerometers, a 3D magnetometer and a 3D gyroscope. A calibrated Shimmer3 IMU, when firmly attached on the limb, can collect precise and accurate data. Each Shimmer3 has a microSD to store the data locally or can stream the data over Bluetooth. Shimmer3 inertial measurement units were used in the exercise data collection process and they were calibrated using Shimmer’s 9DoF Calibration Application (https://www.shimmersensing.com/products/shimmer-9dof-calibration). The IMUs were used with a sampling frequency of 512 Hz along with a calibration range of ±16 g for the 3D low noise accelerometer and a ±2000 dps for the 3D gyroscope. All IMUs used in the process of data capture were calibrated and were securely placed on the right wrist of the participants, as shown in Figure 1b, with the help of an elastic band during the data collection process. The sensor orientation and pictorial representation of the unit attachment on the right wrist are shown in Figure 1a,b respectively.

#### 2.1.2. LME Exercise Set and Experimental Protocol

**Figure A1:**
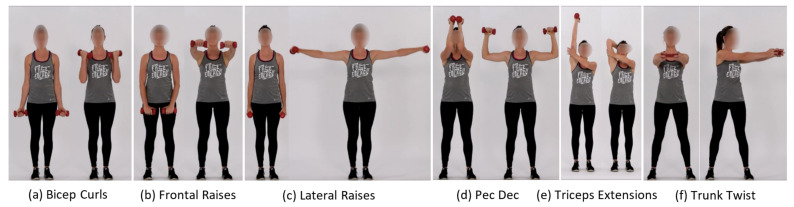
Upper-body LME exercises.

**Figure A2:**
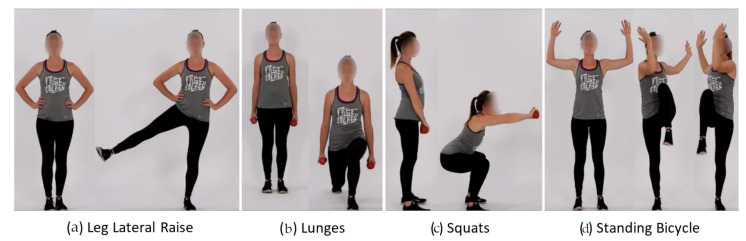
Lower-bodyLME exercises.

Ten LME exercises comprise of six upper-body exercises: Bicep Curls (BC), Frontal Raise (FR), Lateral Raise (LR), Triceps Extension Right arm (TER), Pec Dec (PD), and Trunk Twist (TT); along with four lower-body exercises: squats (SQ), lunges—alternating sides (L), leg lateral raise (LLR), and standing bicycle crunches (SBC). The representative postures for the execution of six upper-body LME exercises are shown in Appendix A, Figure A1 and that of four lower-body LME exercises are shown in Figure A2. A pair of 1 kg dumbbells were used by each participant while performing BC, FR, LR, and PD exercises. A single dumbbell of 1kg was used during TER, TT, L, and SQ. Exercises LLR and SBC were performed without dumbbells. The data from these exercises correspond to ten different classes of exercise. The ten exercises that were used in CVD rehabilitation were either employed a single joint movement effect (BC, FR, LR, PD, TER, and LLR) or employed multiple joint movements (TT, L, SQ, and SBC). Some of these exercises have significantly similar arm movements and hence it was considered of interest to investigate how the models were able to distinguish between these exercises. It was also of interest to see how robust the models were in terms of their capacity to distinguish between the exercise actions in comparison to limb movements that were commonly observed between the exercises. The common limb movements selected for inclusion were side bending, sit-to-stand and sand-to-sit, lean down to lift water bottle or dumbbell kept on the floor, arm-stretching front-straight, lifting folded arm up-word, and body stretching up-word with calf raising for relaxation. These observed common actions have significant similarity in terms of limb movement with that of the exercises. The data corresponding to these common actions together describes the eleventh class of movement.

A total of 76 volunteers (47 males, 29 females, age group range: 20–54 years, median age: 27 years) participated in the data collection process. No participants had any musculoskeletal injury in the recent past which would affect the exercise performance, and all were healthy. Having prior knowledge of the exercise was not a criterion in volunteer recruitment. The study protocols used in data collection were approved by the university research ethics committee [REC Reference: DCUREC/2018/101].

### 2.2. Data Collection for the Insight-Lme Data Set

**Figure A3:**
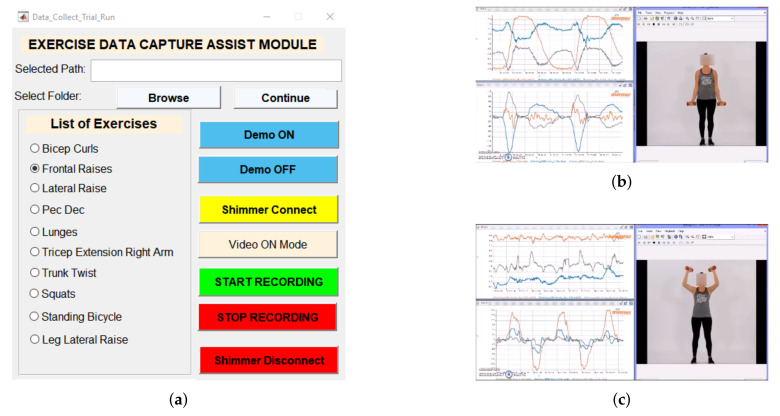
MATLAB–GUI for exercise data capture process. (**a**) Interactive MATH Lab GUI (**b**) Bicep Curls Data Streaming (**c**) Pec-Dec Data Streaming.

The exercise protocol was explained to the participants on their arrival to the laboratory. Each participant underwent a few minutes of warm-up with arm-stretching, leg-stretching and basic body-bending exercises. We developed an exclusive MATLAB–GUI module (https://www.mathworks.com/) [Appendix A, Figure A3a] to collect the data from the participants wearing IMUs via Bluetooth streaming. The “Exercise Data Capture Assist Module” was designed to select a particular exercise, to play demo videos, to initialize and disconnect Shimmer IMUs remotely, to start recording exercise data, to stop recording exercise data and to select a storage path location. The streamed data were stored automatically with participant_ID and the exercise type in the filename, completely anonymizing the details of the participants. We used the Shimmer-MATLAB Instrument driver interface to connect and collect data from multiple Shimmer units, therefore the designed module was capable of recording from multiple participants at any given time.

All consenting participants performed the ten exercises in two sets and the common movements as described in Section 2.1.2. During the constrained set of exercising, the participants performed the LME exercises while observing demonstrative videos on the screen and following the limb movement actions relatively synchronous with the demonstrator in the video. Participants were told to pay particular attention to the following: the initial limb resting position, how to grip the dumbbells (in case the exercise requires the use of dumbbells), the limb movement plane and the speed of limb movement during demo video. The constrained setup facilitated minimal variations in the collected data in terms of planar variations and speed and thus ensuring participants perform exercises at a similar tempo of movement. The participants were asked to perform each exercise for 30 s which resulted in approximately 7 to 8 repetitions. After each exercise, participants were given sufficient time to rest before moving on to the next exercise.

During the unconstrained set of exercising, a timer was used and displayed on the screen. Participants performed the exercises by recalling what they had learned during the constrained performance and were free to execute them for 30 s. The data collected during the unconstrained set corresponds to a variable range of variations from that of exercise data collected from the constrained set of execution. The variations observed were in terms of the plane of limb movement, speed, and the rest position of the limb; these variations were used to mimic macro variations that would typically during home-based exercising.

In addition to the constrained set and the unconstrained set of data collection, participants were instructed to perform the common movements as stated in Section 2.1.2. Inclusion of these non-exercise movements was essential that the built models can distinguish the actions corresponding to the exercise movements from that of non-exercise movements. Participants were asked to perform each of these actions repeatedly for about 30 s. The 5-s instances from each of these actions represent almost one full action and collectively constitutes the eleventh class.

Data collected from both the constrained set and the unconstrained set were class-labelled and stored in ten different exercise folders. An eleventh class-labelled as “others” was created to store the data from all of the common movements. The entire data set is termed the INSIGHT-LME dataset.

### 2.3. The Framework of Different Models

**Figure 2:**
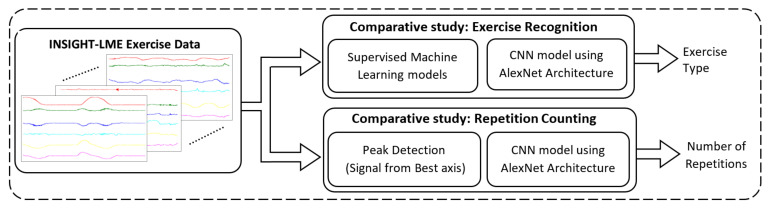
Framework for the comparative study of artificial intelligence models.

Figure 2 represents an overall framework with three major processing blocks. The comparative study aims to find the best possible method from the different AI models for each task in automatic exercise recognition and repetition counting. The first block represents the INSIGHT-LME data set processing and data preparation in terms of filtering, segmentation, 6D vector generations and/or 2D image creation. Data preparation requirements were different for each specific method used in both comparative studies and hence data processing specifics pertaining to the individual method are discussed along with each model below.

The second block represents the comparative study for the exercise recognition task. The exercise recognition task was treated as a multi-class classification task. We compared traditional approaches (supervised ML models) in exercise recognition, with a deep CNN approach based on AlexNet architecture [52]. In supervised ML models, different models were constructed using the four supervised algorithms such as support vector machine (SVM) [53,54], random forest(RF) [55], k-nearest neighbor (kNN) [56] and multilayer perceptron (MLP) [57]. The eight models from these four ML algorithms were studied with and without the dimensionality reduction measures using principal component analysis (PCA) [58]. The best model from the supervised ML was then compared with the deep CNN model to find the best possible method for the exercise recognition task.

The third block represents the comparative study for the repetition counting task. The repetition counting task was treated as a binary classification task followed by a counter to count the repetitions. Again, two different methods were used in repetition counting and the performances were compared to find the best method for repetition counting. We compared traditional signal processing models based on peak detection with a deep CNN approach based on the AlexNet architecture.

#### 2.3.1. Exercise Recognition with Supervised ML Models

**Figure 3:**
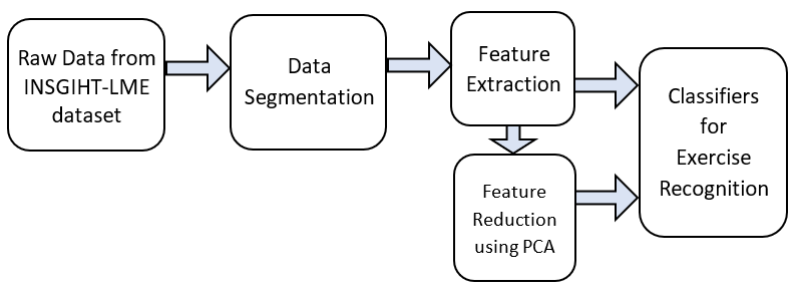
End-to-end pipeline framework for the machine learning models.

Figure 3 illustrates the end-to-end pipeline framework adopted for supervised ML exercise recognition. As discussed in Section 2.3, a total of eight supervised ML models were studied using this framework to classify the 11 activity classes, in which 10-classes were corresponding to the ten LME exercises and the eleventh class “others” for the common movements observed during exercising. The eight supervised ML models were constructed using four algorithms, SVM, RF, kNN, and MLP, either with or without dimensionality reduction using PCA.

##### Data Segmentation

25 s of 3D accelerometer and 3D gyroscope data of each exercise were segmented from the INSIGHT-LME dataset (Section 2.2) retaining class-label information. The segmentation was carried out on all the three sets: training set, validation set and test set from the INSIGHT-LME dataset. 3D accelerometer plots and 3D gyroscope plots for all ten LME exercises are given in Appendix E and Appendix F. The 25 s of 6D segmented data consists of approximately five or six repetitions of an exercise, with each repetition duration lasting approximately 4 s. The segmented data with retained class-label information was used in feature extraction in the next stage.

##### Feature Extraction

Time and frequency features [59] were extracted from the 6D segmented data using an overlapping sliding window method [59]. Three sliding window-lengths of 1 s, 2 s and 4 s were used along with an overlap of 50% in all cases to find an optimum window-length selection in the classifier design. The maximum window-length selection was restricted to 4 s because the length of one complete repetition of an exercise was approximately 4 s.

A vector of 48 features (Table 1), 24 time-frequency features each from the accelerometer and gyroscope, were computed for each sliding window and repeated for every slide. Class-label information was retained. A combined feature set, referred to as “training feature set”, was formed by combining feature vectors from all the exercise classes and the “others” class from the training set. The training feature set is computed for each sliding windows of the 1 s, 2 s and 4 s window-length on the training set of the INSIGHT-LME data set. Similarly, the “validation feature set” and the “test feature set”, is computed on each of the sliding windows of 1 s, 2 s and 4 s input data from the validation set and the test set of the INSIGHT-LME data set, respectively.

Feature sets computed over each sliding window were then used for training, validation and testing of the supervised ML models using four algorithms (SVM, RF, kNN, and MLP) forming a total of 12 classifiers.

##### Feature Reduction Using PCA

To study the effect of dimensionality reduction, principal component analysis (PCA) was used on the feature sets computed from Section 2.3.1 to reduce the overall feature dimensionality of the input vectors to the ML models. Significant principal components, which were having an accumulated variance greater than 99%, were retained [59]. New feature sets corresponding to the training feature set, validation feature set and test feature set were computed using PCA for each of the 1 s, 2 s and 4 s window-length cases. New feature sets with dimensionality reduction using PCA were then used in the training, validation and testing of additional ML models using algorithms (SVM, RF, kNN, and MLP) for each window-length case, resulting in an additional 12 classifiers. Appendix B indicates the PCA computation procedure on the feature vector using the accumulated variance measure.

##### Classifiers for Exercise Recognition

Exercise recognition from the single wrist-worn inertial sensor data for a set of exercises prescribed for cardiovascular disease rehabilitation is a classic classification task using ML or deep learning methods. A total of 24 classifiers were constructed from the feature vectors as explained in Section 2.3.1 and were analyzed for exercise recognition. Each classifier model was constructed using the training set feature vectors, with 10 fold cross-validation using the grid-search method to ensure the models to have optimum hyper-parameters (for SVM models, kernel options between RBF and linear, and model parameters C and gamma values; for kNN models to find the best k-value or number of nearest neighbors; for RF models the number n_estimator or the number of trees to be used in the forest; for MLP the step value α).

All models were first evaluated using the validation set feature vectors to evaluate the following: first, the optimum sliding window-length among all possible selected windowing methods was determined based on the validation accuracy measure. Secondly, to see the effect of dimensionality reduction in ML model performance. Finally, to select the single best-supervised ML model to recognize the exercises based on validation score measure. Furthermore, the best model was evaluated for individual class performance based on statistical measures such as precision, recall and F1-score using Equations (1)–(3) respectively, where TP represents the number of times the model correctly predict the given exercise class, FP represents the number of times the model incorrectly predicts the given exercise class and FN represents the number of times the model incorrectly predicts other than the given exercise class.

(1) $$Precision=\frac{TP}{TP+FP}$$

(2) $$Recall=\frac{TP}{TP+FN}$$

(3) $$F1=\frac{2*Precision*Recall}{Precision+Recall}$$

#### 2.3.2. Exercise Recognition with a Deep CNN Using Alexnet Architecture

**Figure 4:**
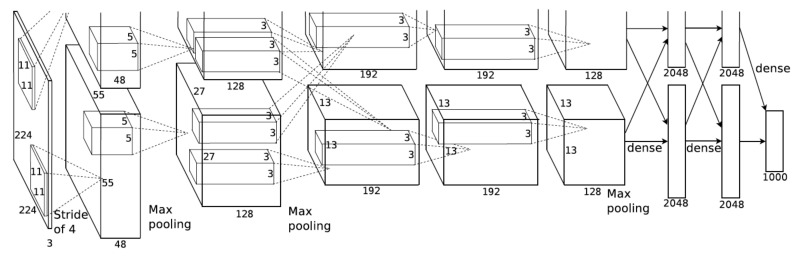
AlexNet architecture [52].

The second method used in the comparative study of the exercise recognition task (Figure 2) was a deep CNN model using the AlexNet architecture (Figure 4) [52]. The AlexNet model consists of eight layers in which five are convolutional layers and three fully connected maximum pooling layers. A rectified linear unit (ReLU) was used as an activation function in each layer and batch normalization were used before passing the ReLU output to the next layer. A 0.4 dropout was applied in the fully connected layers to prevent overfitting of the data. This eight-layered architecture generates a trainable feature map, capable of classifying a maximum of 1000 different classes. The LME exercise recognition task was an 11-class classification task and hence we used a final output layer, a fully connected dense layer, with a SoftMax activation function for the classification of 11-classes. An optimum CNN model was constructed with the best learning rate, optimizer function and loss function using the training data set and was further validated using the validation data set and then tested the model with the test data set from the INSIGHT-LME dataset (Section 2.2). We refer this constructed deep CNN model based on AlexNet architecture as *CNN_Model* hereafter.

##### Data Segmentation and Processing

The *CNN_Model* requires input data in the form of 2D images of size 227 × 227. Data segmentation and processing methods were used to convert the 6D time-series data from the input INSIGHT-LME dataset to 2D images. To compare the results of *CNN_Model* with the ML models discussed in Section 3.2.1, a 4 s windowing method with an overlap of 1 s was used to segment the 6D (3D accelerometer and 3D gyroscope) time-series data and an image of size 576 × 576 with plots of all 6 axes was plotted. An image dataset was generated through data segmentation and processing. This was taken from the entire time-series raw data of the INSIGHT-LME dataset using a 4 s windowing method with a 1 s overlap. The image dataset comprises of 11-classes of image data, among which 10-classes were from the ten LME exercises and the eleventh class from the common movements observed during the exercises. The training set was formed with a total of 43,306 images from 11-classes of data from 46 participants. Similarly, the validation set was formed with 13,827 images from 15 participants and the test set was formed with 14,340 images from 15 participants. Downsampling of input images to 227 × 227 images were further achieved by data augmentation method in the input layer during the model implementation.

##### CNN_Model for the Exercise Recognition Task

An optimum model, *CNN_Model*, was developed using python sequential modelling along with the Keras API [60], a high-end API for TensorFlow [61]. The model constructed here was an optimum model with the best possible optimizer function, good learning rate to achieve better accuracy and with a very good loss function. The model was constructed with the choice of optimizer function among stochastic gradient descent (SGD) [62], Adam [63], and RMSprop [64] and the model was trained with varied learning rates ranging from 1e-03 to 1e-6 values. Also, the model was trained with loss functions such as categorical cross-entropy (CCE) [65] and Kullback–Leibler divergence (KLD) [66]. The best model parameters were selected with an iterative evaluation using a varied number of epochs.

Data augmentations, like resizing of input dataset images and shuffling of input images were achieved using “flow_from_directory” method in “ImageDataGenerator class” from Keras image processing. Since the input images correspond to time-series data, augmentation operations such as shearing, flipping, and rotation tasks were not performed. CNN models were constructed using the training image dataset and validated using the validation image dataset while monitoring the validation loss. A model with a minimum validation loss was saved for each combination of network parameters. The model parameters such as training accuracy, validation accuracy, training loss and validation loss against the number of iterations were obtained and were plotted. The best model with the highest validation accuracy was selected and tested with the test image dataset and the resulting evaluation parameters such as test accuracy and loss measures were recorded. The best model, *CNN_Model*, was then compared with the best model selected from the supervised ML models. A complete list of the architecture parameters can be found in Table A1 in Appendix D.

#### 2.3.3. Exercise Repetition Counting with Peak Detection Method

**Figure 5:**
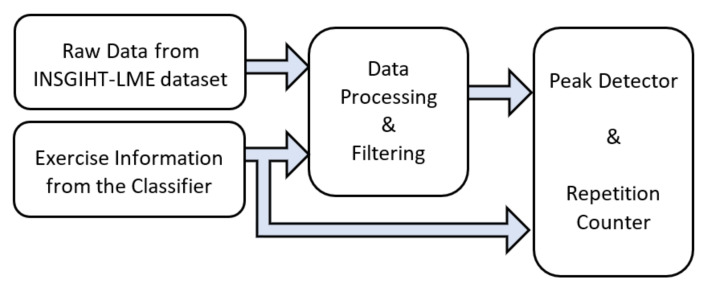
Pipeline for repetition counting using a peak detector.

The first method, among three, investigated for exercise repetition counting was a signal processing method based on peak detection. The concept of the peak detection method [43,59] lies in the identification of the peaks corresponding to the maximum or minimum signal strength of any periodic time-series data. Figure 5 represents the end-to-end pipeline used for peak detection and counting repetitions using peak information. Raw data from the INSIGHT-LME data set corresponds to the 3D accelerometer and 3D gyroscope recordings for limb movement for each of the exercises. Each exercise type exhibits different signal patterns on the different sensor axes and the signal strengths on any given axes are proportional to the plane of limb movement. The periodicity of the signal observed on any significant axis of the sensor was used in the peak detection after completion of the exercise recognition task. Hence, ten peak detectors were used, one for each exercise. The raw data from all the participants from the INSIGHT-LME dataset was used here to count the number of repetitions for each of the exercises. Data processing, filtering, peak detection and counting are discussed in the following section.

##### Data Processing and Filtering

6D time-series data from INSIGHT-LME dataset were the information obtained from each participant while exercising. The signal pattern variations in all the three axes of the accelerometer and the gyroscope represent the significant translatory motion and rotary motion, respectively. While exercising, repetitions are reflected in the periodicity in the signal patterns on these axes of the sensors. The signal amplitude on each axis represents the significance of limb movement in any particular direction. However, these signals were affected by the inherent noise introduced by the sensor. To understand and retrieve these signal variations and to calculate repetitions, the raw data were first processed and filtered.

The first step is to identify a dominant sensor axis for individual exercise and use this signal in peak detection. The dominant sensor axis in the plane of limb movement was evaluated using the mean square values of acceleration measurements from all the three axes of the accelerometer and the mean square values of the rotation rate from all the three axes of the gyroscope.

For each exercise, the observed plane of movement of the right wrist of the participant exercising was matched with the calculated dominant sensor axis using the mean square method (Table 2). Signal plots of 3D accelerometer and 3D gyroscope for all the exercises are shown in Figure A6 of Appendix E and Figure A7 of Appendix F respectively. Dominant-axis signals were smoothed to remove the possible noise using a low pass Savitzky–Golay filter [67]. The Savitzky–Golay filter removes high-frequency noise and has the advantage of preserving the original shape and features of the time-series signal. A window of 1023 samples and a filter order 4 was used.

##### Peak Detection and Repetition Counting

**Figure 6:**
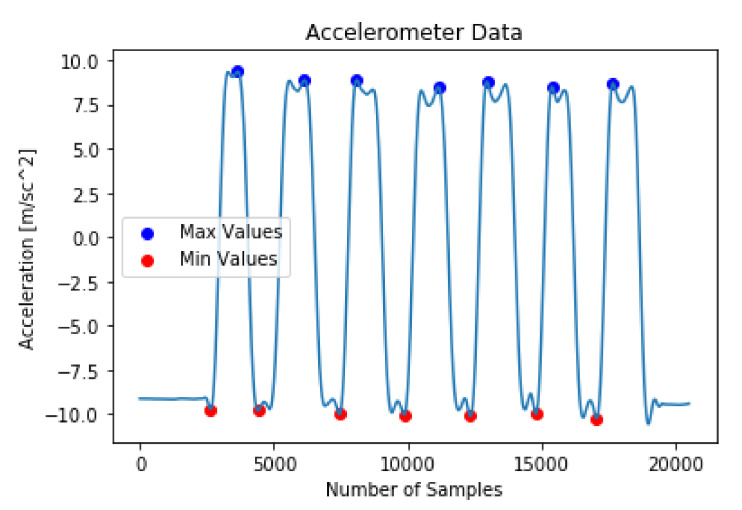
An example of repetition counting for Bicep Curls on the filtered dominant signal from the x-axis of the accelerometer sensor.

The peak detector detects both positive peak and negative peak values from the input time-series signal using a threshold value. For individual exercise type, the threshold value was unique and was calculated using the dominant-axis signal information [43]. Two cut-off points were calculated using the threshold value, an upper threshold point and a lower threshold point. Using these two cut-off values the peak detector determined the subsequent max and min values from the input wearable sensor signal. A max–min pair constitutes a repetition count and used as an increment in the repetition counting process. Figure 6 represents the filtered accelerometer x-axis signal for the Bicep Curls with the positive and negative peaks marked using a peak detector. Accelerometer x-axis was the dominant signal information for Bicep Curls (Table 2). A total of ten different peak detectors were used, one for each exercise.

#### 2.3.4. Exercise Repetition Counting with a Deep CNN Using Alexnet Architecture

**Figure 7:**
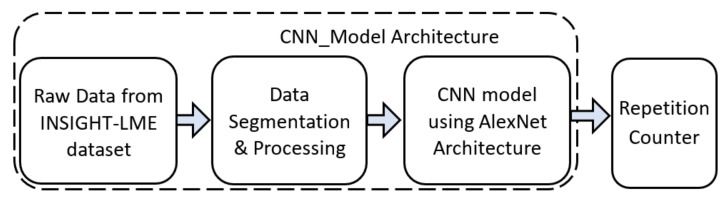
Pipeline for repetition counting using *CNN_Model*.

The second approach investigated for repetition counting was a deep CNN model, based on the AlexNet architecture (*CNN_Model*). We compare a single deep CNN model for the repetition counting task of all the exercises as opposed to the use of multiple CNN models as used in [27]. Figure 7 illustrates the pipeline used for the repetition counting task using the *CNN_Model* as a binary classifier along with an additional repetition counter block. Inspired by the signal processing approach to the repetition counting, *CNN_Model* uses the peak information from the signals. However, the *CNN_Model* uses a binary classifier for the repetition counting instead of 11-class classifier as in the case of the exercise recognition task (Section 2.3.2). The output of the binary classifier using the *CNN_Model* was given to a repetition counter which counts the total repetitions for any given exercise.

##### Data Segmentation & Processing

Using the dominant-axis information and the image dataset created with a 4 s sliding window from Section 2.3.2 and we created new binary target label information. New binary target class-label information was generated using a grid of 50% width of the image and if the peak of the dominant-axis signal plot in the image lies on the left half of the vertical axis of the grid then the image was labelled with “Peak” (“1”) otherwise, the image was labelled with “NoPeak” (“0”). The binary class-label were applied to the training, validation and test image data-sets.

##### CNN_Model as a Repetition Counter

**Figure 8:**
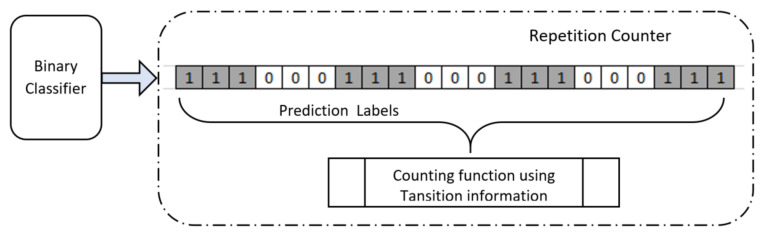
Repetition Counter.

Models were trained with the training dataset of the newer image dataset with binary class-label information and validated with the validation results. *CNN_Model* was built to have optimum parameters with variation in learning rate and selection of optimizer as discussed in Section 2.3.2. We used a binary cross-entropy loss function while training all models and the best model was selected based on the validation score evaluation. Repetition counting was done by testing a sequence of 43 images corresponding to a 25 s exercise data. The predicted result, from the model, on each image of the sequence, was recorded and used in the repetition counter. A repetition counter counts the total number of transitions from “Peak” to “NoPeak“ (“1” to “0”) and from “NoPeak” to “Peak” (“0” to “1”). The total repetition count corresponds to half the number of total transitions from the prediction labels (Figure 8).

## 3. Results

### 3.1. Results of Data Sampling

Among 76 participants, 75 people participated both in the constrained set and unconstrained set of data collection. However, one participant performed only the constrained set. Only a few participants had not performed all the exercises. The collected data set was an overall well-balanced dataset and Table 3 indicates the participation summary for each exercise under the constrained set and the unconstrained set of data capture. The data set was then segregated and stored into three different sets: the training set, the validation set and the test set, and was used in all model building. The data from 46 participants were used in the training set and the data from 15 participants were used in both the validation set and the test set.

#### Summary of Data Sampling

No public dataset was available with a single sensor wearable device specifically for the LME exercises used in CVD rehabilitation which could be used on mHealth platforms. We created the INSIGHT-LME dataset from 76 willing participants performing LME exercises in two sets. Data collected from the participants wearing a single wrist-worn wearable device under the supervision of health experts from the sports clinic and with the guidance from clinical staff. The new dataset will encourage further research in the field of application using a single wrist-worn inertial sensor in exercise-based rehabilitation.

### 3.2. Results for the Exercise Recognition Task

#### 3.2.1. Experimental Results of Exercise Recognition with Supervised ML Models

A total of 24 classifiers were constructed using three sliding windowing methods with four supervised ML algorithms with and without dimensionality reduction using PCA. These models were constructed using a 10-fold cross-validation method. The SVM models were constructed using One-Vs-Rest multi-class classifier and were designed to have optimum hyper-parameters using a grid-search method with 10-fold cross-validation. The values, C = 100, gamma = 0.01 and RBF kernel were found to be the optimum hyper-parameters for all the 6 SVM classifiers. For all the 6 kNN models, k = 1 found to be the optimum value and for all the 6 RF models, n_estimator = 10 found to be the optimum value. Similarly, for all the 6 MLP classifiers the step value, α = 1, was optimum over a range of 1e-5 to 1E+3 on a logarithmic scale.

Selection of suitable sliding window-length was done based on the validation results using the validation feature set. While the training score indicates the self-classifying ability of the model, the validation score helps in accessing the suitability of any model deployment on the unseen data. The training and validation scores for all the 24 classifiers segregated with the corresponding window-length are shown in Table 4. Validation score measures for the models built using 1 s window-length were less compared to the validation score measures of the models built using 2 s and 4 s window-length for all the four (SVM, MLP, kNN, and RF) models with and without PCA. Therefore, all the models built using 1 s are not selected. In addition, in terms of validation score measure, the performance of the supervised ML models built using 4 s window-length was showing 1% to 2% improvement when compared with the models built with a window-length of 2 s. Therefore, the eight supervised ML models constructed using 4 s sliding window-length (with and without PCA) were retained for further comparison. All the eight models, from 4 s window-length, were tested with the same test set data using the test set features to find a single best-supervised classifier for exercise recognition.

Test-score measures for eight selected supervised ML classifiers are recorded in Table 4. The SVM model without PCA was found to be the single best performing model with a test score of 96.07%. The SVM model with PCA was found to be the second-best model with a test score of 95.96%. Furthermore, a common observation can be drawn between the models constructed with and without PCA. For all the four supervised ML algorithms (SVM, MLP, kNN, and RF) the test-score measures have not improved with the dimensionality reduction. The SVM model without PCA was selected as the best-supervised ML model and was further evaluated to find the performance on individual exercises. The SVM model performance for each exercise, in terms of precision, recall and F1-score measures, were tabulated in Table 5.

From the performance evaluation of the SVM classifier on individual exercises (Table 5), we can conclude that using a single wrist-worn inertial sensor in the CVD rehabilitation process, we could achieve the exercise recognition with an overall recall rate of 96.07%. This result is very important as the set of LME exercises used in this study are not only single joint upper-body exercises but also have exercises with multi-joint lower-body exercises. For the upper-body LMEs, measured overall precision was 96.41%, overall recall was 96.77% and overall F1-score was 96.59% and for the lower-body LMEs, measured overall precision was 95.96%, overall recall was 96.29% and overall F1-score was 96.12%.

**Figure 9:**
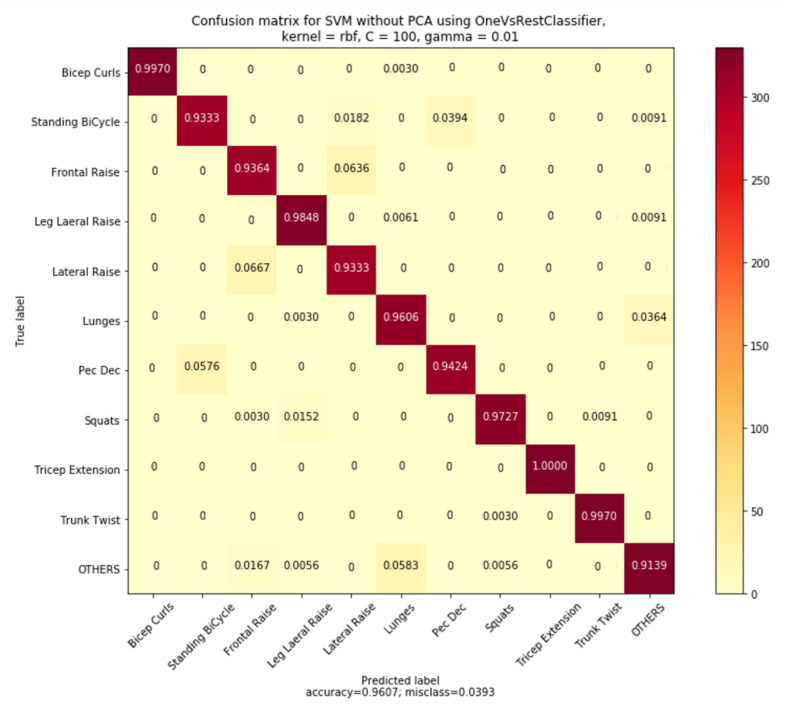
Normalized confusion matrix for the SVM model.

**Figure A5:**
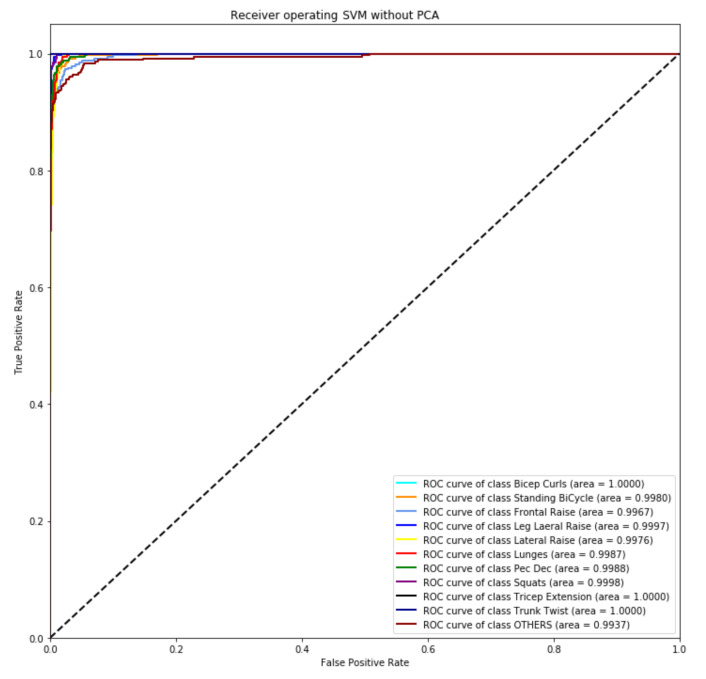
Receiver operating characteristic of the SVM model.

**Figure A6:**
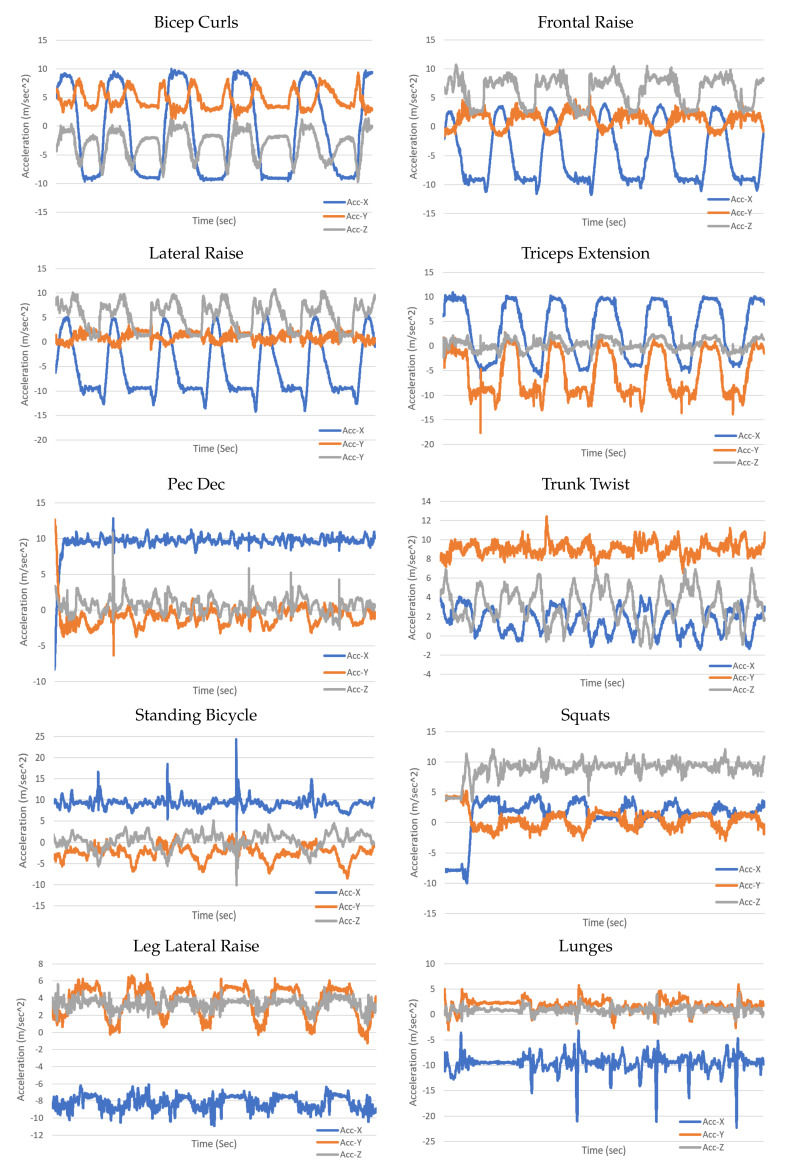
25 s 3D accelerometer plesignal plots for all exercises.

**Figure A7:**
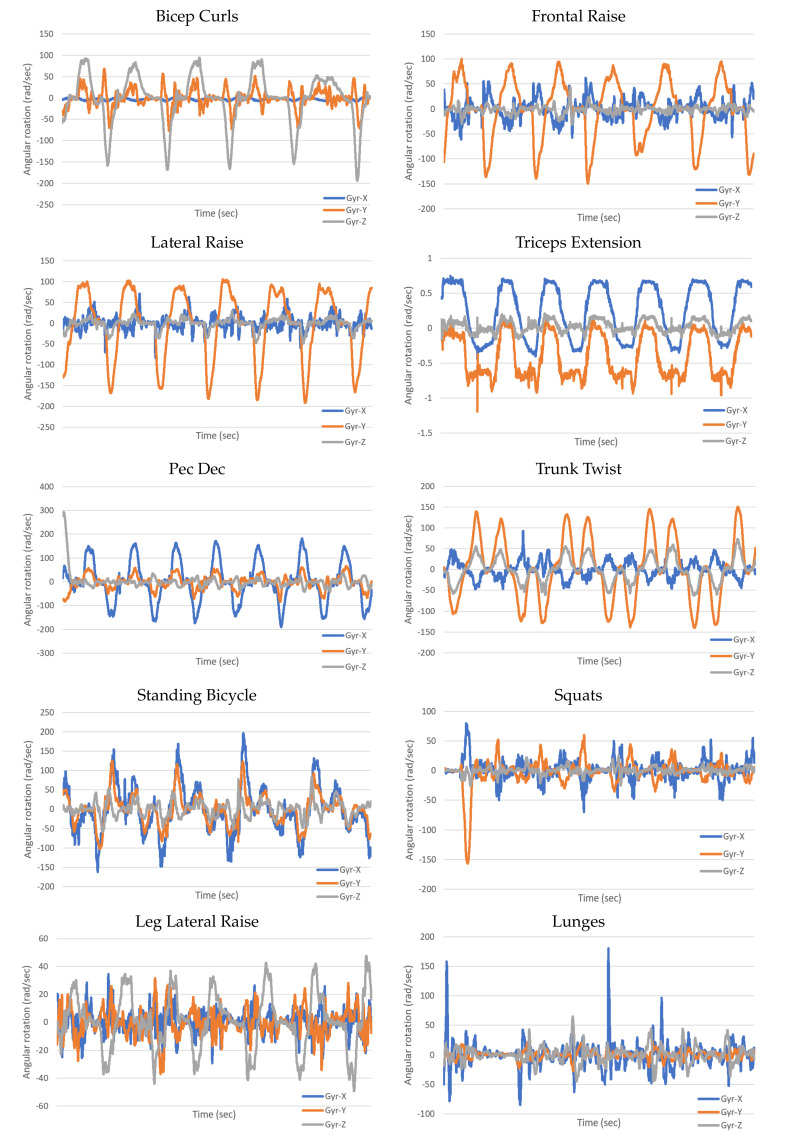
25 s 3D gyroscope signal plots for all exercises.

The model’s normalized confusion matrix plot representing the confusions among the exercises are plotted and shown in Figure 9. Confusions among the exercises with similar wrist-movement actions were evident from the confusion matrix plot and are discussed here. The first observed confusion was between two upper-body LMEs, the Frontal raises (FR) and the Lateral raises (LR), and 6.36% of the FR exercises were confused with that of the LR while 6.67% of the LR exercises were confused with that of FR. In both FR and LR exercises, raising the hands straight was commonly observed with significant movements on the plane of the accelerometer x-axis direction. However, the wrist-movement actions were different for FR from that of LR only during the movement from the initial resting position. The second observed confusion was between the exercises Pec Dec (PD) and the Standing bicycle crunches (SBC). A 3.94% confusion was observed in SBC from PD, whereas a 5.76% of PD was getting confused with SBC. The wrist rotary movements in the plane of the gyroscope y-axis direction were similar for these SBC and PD exercises. The third observation was for the lower-body LME exercise Lunges were getting confused with the common movements (others) and a 3.64% confusion was observed. However, the common movements (others) were confused with Lunges with a 5.83% confusion. AUC-ROC plot for individual exercise recognition is given in Appendix C (Figure A5).

#### 3.2.2. Experimental Results of CNN _Model

The *CNN_Model* with Adam optimizer, a learning rate 1e-4 with KLD loss function was the best model with a training score of 99.96% and a validation score of 94.01%. The model was further evaluated using the test set image dataset and measured an overall test score of 96.90%. This overall test-score measure was almost 1% improved in comparison with the SVM model, the best performing supervised ML model (Section 3.2.1). The performance of *CNN_Model* for the individual exercises was evaluated and the statistical parameters measures like precision, recall and F1-score for each exercise were tabulated in Table 6. These test-score measures in terms of precision, recall and F1-score for the individual exercise recognition of the CNN model with AlexNet architecture (Table 6) were improved in comparison to the test-score measures obtained from the SVM model (Table 5).

**Figure 10:**
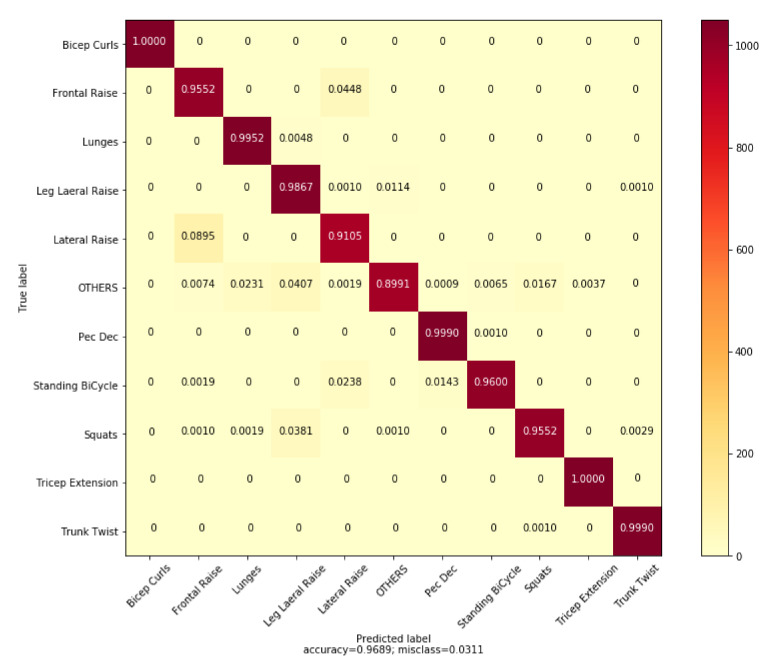
Normalized confusion matrix for CNN model with AlexNet architecture.

**Figure 11:**
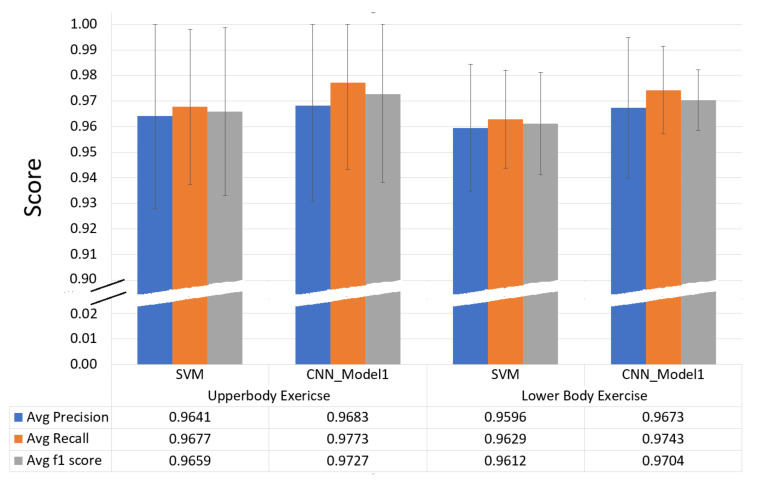
Statistical parameter comparison for *CNN_Model* and SVM models.

Figure 10 represents the normalized confusion matrix for the *CNN_Model*. The values on the main diagonal representing recall or sensitivity of the model to the individual exercises. The improvement of overall recall rate by almost 1% can be seen from the amount of less confusions among exercises from the confusion matrix. Major confusions between the exercises are improved compared to the SVM model. For example, confusion between LR and FR is reduced to 4% in comparison with 6% in the SVM model. Similarly, confusion among SBC and PD is reduced to almost 1% in comparison with 5% in SVM model. Overall performance comparison of the SVM model and *CNN_Model* for upper-body and lower-body exercises along with standard deviation measure is shown in Figure 11. The *CNN_Model* outperformed the SVM model in both the upper-body LME exercises and the lower-body LME exercises.

##### Summary of Comparative Study of Models for the Exercise Recognition Task

Our first study was to find a single best model for the exercise recognition by comparing traditional supervised ML methods with a deep learning method. We studied the supervised ML models using SVM, RF, kNN and MLP with and without dimensionality reduction using PCA. Also, we studied a deep CNN model based on the AlexNet architecture. We selected the supervised ML models with 4 s window-length based on validation score. The models with PCA were observed with lower test-score performance compared to the models without PCA. SVM model without PCA was found to be the single best performing supervised ML model with an overall test accuracy measure of 96.07%. In addition, the deep CNN model, *CNN_Model*, had an overall test accuracy measure of 96.89% and found to be the single best performing model for the exercise recognition task. Beside overall test-score measure, overall precision, recall and F1-score measures of the *CNN_Model* outperformed the SVM model both in the upper-body and the lower-body LME exercise recognition tasks.

### 3.3. Results for the Exercise Repetition Counting Task

#### 3.3.1. Experimental Results of Repetition Counting Using Peak Detectors

All the input data signals from the INSIGHT-LME dataset were used in testing to evaluate the overall performance of the peak detectors. The number of error counts, i.e., the difference between the actual number of repetition counts and the number of detected counts, was recorded in each case. Table 7 shows the results of repetition counting for individual LME exercise in terms of the number of errors with that of the actual count using peak detection method.

The table also indicates the total number of subjects that were used in testing each exercise. The repetition error counts were indicated by the columns “Error counts’’ or “e|X|’’ where “e|X|’’ indicates the number of exercise sets with ‘|X|’ repetition error count. ‘|X|’ represents the absolute error count in terms of 0, 1, 2, or more than 2 errors. The peak detector method used for the repetition counting performed better for upper-body exercises like BC, FR, LR and TER in comparison to the repetition counting of the lower-body exercises. For example, from Table 7, for Bicep Curls, an upper-body LME exercise, repetition counting without any error were reported for 144 instances among 151 subject trials. However, for 7 subject trials, ±1 error count was reported.

#### 3.3.2. Experimental Results of Repetition Counting Using *CNN _Model*

The optimization of parameters was selected based on lowest validation loss measures and the optimum *CNN_Model* for the repetition counting task was with Adam optimizer and with a learning rate of 1e-5 The model was further tested with the test dataset images. The test data set corresponds to the data from 15 participants and each exercise was performed twice by each participant resulted in a total of 30 exercises data for each exercise.

Table 8 shows the result of the repetition counting for individual LME exercises in terms of the number of errors with that of the actual count. The overall performance of the model in the repetition counting using a single AlexNet architecture-based *CNN_Model* was very accurate for most of the upper-body LME exercises. However, for the lower-body exercises, the repetition count performance for LLR was 80% and was better compared to the performance with other lower-body exercises. For the lunges, the model performance was poorest in the repetition counting.

The performance of the model for the upper-body LMEs like FR and LR, it was 100%. For other upper-body LMEs like BC, TER, PD correct counting was 96.67%. In the case of LLR, a lower-body LME exercise, the correct counting was 80%. For other exercises the performances of the model with zero error count were poor. However, the overall count performance of *CNN_Model* was improved for most of the exercises, when compared to the repetition counting using the signal processing model (Table 7). Thus, the repetition count performance of the *CNN_Model* five out of ten exercises was >95% and for four exercises it was in the rage of 60~80%. Also, it was observed that the overall performance by the *CNN_Model* in repetition counting of the upper-body LMEs was >92%. However, the *CNN_Model* for repetition counting suffered in the case of Lunges, a lower-body LME exercise. In total, with a tolerance of ±1 count error, the performance of the CNN model was accurate in 90% or repetition sets.

##### Summary of Comparative Study of Models for the Exercise Repetition Counting Task

We studied two different methods for the exercise repetition counting task. First, the signal processing-based approach or peak detectors and the second, *CNN_Model* using the AlexNet architecture. We designed ten different peak detectors based on the dominant sensor-axis signal information, one for each exercise. The peak detector was a dependent model and works as a sequential block after a particular exercise recognition. This brings inherited latency of sequential processing. Signal processing method found to be more accurate method in terms of accurate counting of repetition counts including the lower-body LME exercise, lunges. However, the models were under-weighed because of two facts: first, the requirement of ten different peak detectors one for each exercise recognition and second, the method was a follow-up sequential block with the dependency on the completion of the exercise recognition. However, the *CNN_Model* was a single deep CNN model used for the repetition counting which can count the repetition without waiting for the completion of exercise recognition. To the best of our knowledge, use of a single deep CNN model for the repetition counting among a varied range of exercises is novel. With a tolerance of ±1 count error, the performance of *CNN_Model* was accurate in 90% or repetition sets.

## 4. Discussion

In this paper, we compared models to find a single best artificial intelligence model for automatic recognition and repetition counting in LME exercises used in CVD rehabilitation program using single wrist-worn device. We found a deep CNN model constructed using state-of-the-art AlexNet architecture is the best model for the exercise recognition and repetition counting in terms of accuracy measure. The deep structure associated with the AlexNet learns better compared to the handcrafted feature learning associated with supervised ML models. Considering only supervised ML models, the SVM model without PCA was best for recognizing the set of LME exercises. In addition, we demonstrated a novel method of using a single CNN model for all the exercise repetition counting. We generated a novel dataset comprising of data for ten LME exercises and six common movements observed between the exercises (INSIGHT-LME).

Though our work was carried out on the LME dataset generated during this study, we would like to compare our findings with the outcome of recent relevant research works in the area of exercise-based rehabilitation. First study, Soro et al. [27] examined exercise recognition and repetition counting using deep CNN models. The work [27] was carried on a set of ten Cross-Fit exercises and makes use of two sensors one on a foot and one on hand, and uses a single deep CNN for the exercise recognition task, designed from scratch. However, this study of exercise recognition is only on the exercise movements with an assumption of only exercising environment and does not consider any other commonly observed non-exercise movements between exercises. The data was recorded from accelerometer, gyroscope and orientation sensor giving rice to 9D data from each sensor. The work [27] reports a test accuracy measure of 97% using a single hand-worn sensor device. In contrast, our model for the exercise recognition, *CNN_Model*, uses 6D data, (accelerometer and gyroscope) and reports 96.89% test accuracy score which is almost same. However, our model was trained to recognize the exercises considering an additional eleventh class (“Others”), with non-exercise movement data along with the ten exercise class data. In addition, the work [27] also studies exercise repetition counting and uses ten different CNN models, one each for the repetition counting of each type of exercise. The individual models built for repetition counting were sequential blocks, which work only after the exercise recognition. They achieved ±1 error among 91% observed sets. In contrast, we built a single CNN model as it eliminates the need for an exercise specific repetition counter and reduces the dependency on the total number of resources required in repetition computation. Our single CNN model was capable of counting repetitions from all the exercises distinguishing from non-exercise actions. We have achieved repetition counting with ±1 error count on 90% observed data-sets. It appears that our study is novel in using a single CNN model for the exercise repetition counting.

A second study, by Um Terry et al. [23], uses the PUSH data set for the exercise motion classification using a single CNN model for the automatic rehabilitation and sport training. The data set is a private dataset provided by PUSH Inc., collected using PUSH, a forearm-worn wearable device for measuring athletes’ exercise motions. The study uses a subset of exercise data for gym-based exercising from athletes and uses 50 exercises for their classification study. The 9D data comprises of accelerometer, gyroscope and orientation sensors. Similar to our study of generating 2D image patterns from the raw 6D data, their study uses the image patterns obtained using 9D data. However, their study differs in the input image data set formation, where the input image data set was formed using 3 different rectangular grids of varied sizes. Their CNN model resulted in an overall test accuracy measure of 92.1% for the exercise classification. They also found that a CNN model with 3 levels performed better than with 2. In our study, our deep CNN model uses AlexNet architecture which is with a deep model with a depth of 8 levels. The additional levels with the AlexNet may have contributed to the improved accuracy (96.89%).

A third study, by Zheng-An et al. [35], used a multipath CNN model for sensor-based rehabilitation exercise recognition. The study made use of a CNN model based on Gaussian mixer models on the wearable sensor data as first channel path information and second CNN to calculate state transition probability using Lemple–Ziv–Welch coding. A third CNN was used on the combined two channel information for the exercise classification. The study used four rehabilitation exercises using an internet of things (IoT)-based wearable sensor and the data used information from the accelerometer, gyroscope, and magnetometer. The four exercises were stretching exercises while sitting on a chair. The study reported a test accuracy measure of 90.63% using the multipath CNN model. This approach of multipath CNN-based learning is a combination of feature learning from different methods and is different to our approach as we used a single deep CNN model for the exercise recognition and repetition counting. However, our study also differs with [35] in that we employed a greater number of exercises, more diverse limb movements and larger limb movements in the exercises.

## 5. Conclusions

While our study and those of Soro et al. [27], Um Terry et al. [23] and Zheng-An et al. [35] used different exercises and different data-sets, they all have tried to address exercise-based rehabilitation using deep learning models. The present state-of-the-art deep CNNs appear to show higher accuracy measures in comparison to the supervised ML models due to the ability of deep CNNs to learn a higher number of features in comparison to the associated handcrafted feature learning with ML models. Our work also shows that it is possible to use a single CNN model to count exercise repetition, with very little loss in accuracy. This may be beneficial in reducing the dependency on the total number of resources required in repetition computation in the case of multiple exercise evaluation.

We studied exercise recognition and repetition counting using single CNN models; future research should explore their use in providing qualitative feedback on the ‘correctness’ of the movement technique by observing the variations in the exercise execution in comparison to an ‘acceptable’ technique. Finally, we have studied the tasks of exercise recognition and repetition counting in an offline mode with a windowing method. These approaches can be further studied in terms of their time complexity to examine their implementation on miniaturized wearable devices. 

## Figures and Tables

**Table 1:** List of time and frequency domain features computed from the 3D accelerometer and 3D gyroscope data.

Number of Features	Feature Description from Accelerometer and Gyroscope
12	Minimum and Maximum from each axis
12	Mean and Std Deviation from each axis
6	RMS values from each axis
6	Entropy value computed from each axis
6	Energy from the FFT coefficient from each axis
6	Pearson correlation coefficients between the axis

**Table 2:** The sensor and the dominant-axis information for Individual LME Exercises.

Exercise Type		Acronym	Sensor Used & Dominant Axis
**Upper-Body** **LME Exercises**	Bicep Curls	BC	Accelerometer: X-Axis
	Frontal Raises	FR	Accelerometer: X-Axis
	Lateral Raises	LR	Accelerometer: X-Axis
	Triceps Extension Right	TER	Accelerometer: X-Axis
	Pec Dec	PD	Gyroscope: X-Axis
	Trunk Twist	TT	Gyroscope: Y-Axis
**Lower-Body** **LME Exercises**	Standing Bicycle Crunch	SBC	Gyroscope: X-Axis
	Squats	SQ	Accelerometer: X-Axis
	Leg Lateral Raise	LLR	Accelerometer: Y-Axis
	Lunges	L	Accelerometer: X-Axis

**Table 3:** Data capture participation summary.

Exercise Type	Exercise Acronym	Number of Participants	
		Constrained Set	Unconstrained Set
Upper-Body LME exercises	BC	76	75
	FR	76	75
	LR	76	74
	TER	76	75
	PD	75	74
	TT	76	75
Lower-Body LME exercises	SBC	75	74
	SQ	73	73
	LLR	75	74
	L	73	75
Others	OTH	76	75

**Table 4:** Classifier performance comparison over varied window-lengths.

Window Length	Classifiers	Scores (without PCA)			Scores (with PCA)		
		Training	Validation	Test	Training	Validation	Test
**1 s**	SVM	0.9735	0.8559	Models Not Selected	0.9674	0.8525	Models Not Selected
	MLP	0.9232	0.8190		0.9041	0.8041	
	kNN	0.9390	0.8248		0.9307	0.8227	
	RF	0.9925	0.8165		0.9898	0.8179	
**2 s**	SVM	0.9907	0.8906	Models Not Selected	0.9875	0.8816	Models Not Selected
	MLP	0.9690	0.8615		0.9568	0.8475	
	kNN	0.9715	0.8571		0.9613	0.8520	
	RF	0.9956	0.8607		0.9850	0.8439	
**4 s**	SVM	0.9974	0.9171	0.9607	0.9965	0.9089	0.9596
	MLP	0.9961	0.8709	0.9328	0.9939	0.8709	0.9347
	kNN	0.9944	0.8848	0.9415	0.9845	0.8828	0.9388
	RF	0.9995	0.8905	0.9467	0.9994	0.8670	0.9333

**Table 5:** Performance evaluation measures of SVM Classifier on individual exercises.

Exercise Type		Acronym	Precision	Recall	F1-Score
**Upper-Body** **LME exercises**	Bicep Curls	BC	1	0.9970	0.9985
	Frontal Raise	FR	0.9142	0.9364	0.9252
	Lateral Raise	LR	0.9194	0.9333	0.9263
	Triceps Extension	TER	1	1	1
	Pec Dec	PD	0.9599	0.9424	0.9511
	Trunk Twist	TT	0.9910	0.9970	0.9940
**Lower-Body** **LME Exercises**	Standing Bicycle Crunches	SBC	0.9419	0.9333	0.9376
	Squats	SQ	0.9907	0.9727	0.9817
	Leg Lateral Raise	LLR	0.9760	0.9849	0.9804
	Lunges	L	0.9296	0.9606	0.9449
**Common** **Movements**	Others	OTH	0.9481	0.9139	0.9307

**Table 6:** Performance evaluation measures of *CNN_Model*.

Exercise Type		Acronym	Precision	Recall	F1-Score
**Upper-Body** **LME exercises**	Bicep Curls	BC	1	1	1
	Frontal Raise	FR	0.9052	0.9552	0.9296
	Lateral Raise	LR	0.9273	0.9105	0.9188
	Triceps Extension	TER	0.9962	1	0.9981
	Pec Dec	PD	0.9850	0.9990	0.9920
	Trunk Twist	TT	0.9962	0.9990	0.9976
**Lower-Body** **LME Exercises**	Standing Bicycle Crunches	SBC	0.9921	0.9600	0.9758
	Squats	SQ	0.9814	0.9552	0.9681
	Leg Lateral Raise	LLR	0.9209	0.9867	0.9526
	Lunges	L	0.9748	0.9952	0.9849
**Common** **Movements**	Others	OTH	0.9868	0.8991	0.9409

**Table 7:** Number of error counts in the repetition using Peak Detector Method.

Exercise Type	Exercise	Acronym	Total Subjects	Error Count			
				e|0|	e|1|	e|2|	e>|2|
**Upper-Body** **LME Exercises**	Bicep Curls	BC	151	144	7	0	0
	Frontal Raises	FR	151	140	11	0	0
	Lateral Raises	LR	150	141	9	0	0
	Triceps Extension Right	TER	152	143	9	0	0
	Pec Dec	PD	149	120	8	3	18
	Trunk Twist	TT	151	128	14	5	4
**Lower-Body** **LME Exercises**	Standing Bicycle Crunch	SBC	149	132	8	4	5
	Squats	SQ	146	63	11	6	66
	Leg Lateral Raise	LLR	149	73	10	18	48
	Lunges	L	147	11	9	13	114

**Table 8:** Number of error counts in the repetition using *CNN_Model*.

Exercise Type	Exercise	Acronym	Total Subjects	Error Count			
				e|0|	e|1|	e|2|	e>|2|
**Upper-Body** **LME Exercises**	Bicep Curls	BC	30	29	1	0	0
	Frontal Raises	FR	30	30	0	0	0
	Lateral Raises	LR	30	30	0	0	0
	Triceps Extension Right	TER	30	29	0	0	1
	Pec Dec	PD	30	29	0	0	1
	Trunk Twist	TT	30	19	5	3	3
**Lower-Body** **LME Exercises**	Standing Bicycle Crunch	SBC	30	18	9	1	2
	Squats	SQ	30	19	10	0	1
	Leg Lateral Raise	LLR	30	24	3	1	2
	Lunges	L	30	3	6	11	10

**Figure A4:**
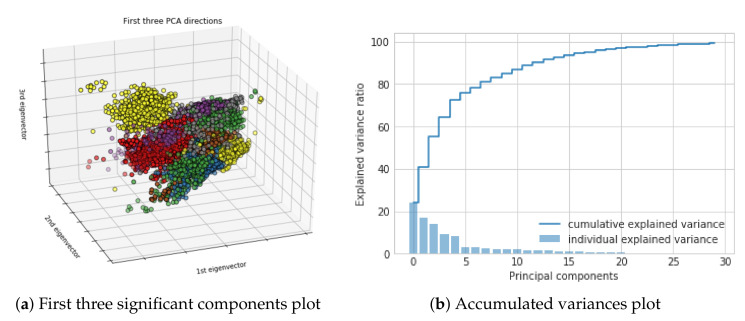
PCA plots for the training feature set for a 4 s sliding window (**a**) 3D plot of the first three significant components, (**b**) Accumulated variances plot.

## Appendix B. Representation of PCA Computation of Time-Frequency Feature Vectors

PCA plots based on 30 traits measured on 11-classes of movements (10-classes of exercise movements and an ’other class’) from the INSIGHT-LME dataset shown in Figure A4. First three significant components are given in Figure A4a. An accumulated variance plot is shown in Figure A4b.

## Appendix C. Receiver Operating Characteristic of the SVM Model (AUC-ROC Plot)

Performance measurement or the capability of the classifier models were represented using the area under the curve plot or also known as the receiver operating characteristic (AUC-ROC) curve plots. The AUC-ROC curves are plotted with the true positive rate (TPR) on the y-axis against the false positive rate (FPR) the x-axis. Figure A5 represents the AUC-ROC plot for the SVM classifier without PCA and a minimum AUC value of 99.67% for FR to a maximum of 100% for BC, TT and TER.

## Appendix D. Model Architecture for *CNN _Model*

CNN Model architecture used in the exercise recognition task for *CNN_Model* is given in Table A1 and represents the number of layers and the parameters used. The same model with only output layer variation is used in the repetition counting task

**Table A1:** All architecture parameters for *CNN_Model*. CL: Convolution Layer, DL: Dense Layer.

Layer	Value	Parameters
Input Layer	227 × 227 × 3	0
Convolution Filters CL1	96	34,944
Kernel Size CL1	(11, 11)	-
Strides CL1	(4, 4)	-
Pooling PL1	(3, 3)	0
Strides PL1	(2, 2)	-
Convolution Filters CL2	256	614,656
Kernel Size CL2	(5, 5)	-
Strides CL2	(1, 1)	-
Pooling PL2	(3, 3)	0
Strides PL2	(2, 2)	-
Convolution Filters CL3	384	885,120
Kernel Size CL3	(3, 3)	-
Strides CL3	(1, 1)	-
Convolution Filters CL4	384	1,327,488
Kernel Size CL4	(3, 3)	-
Strides CL4	(1, 1)	-
Convolution Filters CL5	256	884,992
Kernel Size CL5	(3, 3)	-
Strides CL5	(1, 1)	-
Pooling PL3	(2, 2)	0
Strides PL3	(2, 2)	-
Dense DL1	4096	4,198,400
Dropout DL1	0.4	0
Dense DL2	4096	16,781,312
Dropout DL2	0.4	0
Dense DL3	1000	4,097,000
Dropout DL3	0.4	0
Batch Normalization CL1, CL2, CL3, CL4, CL5, DL1, DL2, DL3	Yes	384 + 1024 + 1536 + 1536 + 1024 + 16,384 + 16,384 + 4000
Activation function CL1, CL2, CL3, CL4, CL5, DL1, DL2, DL3	ReLU	0
Activation function DL2	SoftMax	0
**Total Parameters**	**:**	**28,877,195**
**Trainable Parameters**	**:**	**28,856,059**
**Non-trainable Parameters**	**:**	**21,136**

## Appendix E. 3D Accelerometer Raw Data Signal Plots for All Exercises

3D Accelerometer raw data signal plots for all the 10 LME exercises corresponding to the data from the wrist-worn sensor of one participant is shown in Figure A6.

## Appendix F. 3D Gyroscope Raw Data Signal Plots for All Exercise

3D Gyroscope raw data signal plots for all the 10 LME exercises corresponding to the data from the wrist-worn sensor of one participant is shown in Figure A7.
